# Assessment of prognostic LV parameters with CMR in hypertrophic cardiomyopathy: impact of the papillary muscles

**DOI:** 10.1186/1532-429X-14-S1-P160

**Published:** 2012-02-01

**Authors:** Frank Gommans, Jeanette Bakker, Etienne G Cramer, Michael J Kurvers, Marc A Brouwer, Freek W Verheugt, Marcel J Kofflard

**Affiliations:** 1 Cardiology, Radboud University Nijmegen Medical Centre, Nijmegen, Netherlands; 2 Radiology, Albert Schweitzer Hospital, Dordrecht, Netherlands; 3 Cardiology, Albert Schweitzer Hospital, Dordrecht, Netherlands

## Background

Hypertrophic cardiomyopathy (HCM) is the most common inheritable heart disease, characterized by marked left ventricular hypertrophy in the absence of a disease that can cause hypertrophy to such extent. Both LV ejection fraction (EF) and LV mass have proven to be prognosticators in HCM.

The literature is inconsistent with respect to in- or exclusion of papillary muscles (PMs) to LV-mass, which might influence volumetric results. PMs might be part of the hypertrophic process and substantially contribute to LV mass.

We investigated the impact of in- and exclusion of the PMs to LV mass in the assessment of prognostic LV parameters in HCM.

## Methods

Three groups of subjects were included:

1) HCM patients: phenotype +, genotype + (n = 27)

2) HCM patients: phenotype +, genotype - (n = 36)

3) preclinical HCM mutation carriers: phenotype -, genotype + (n = 10).

Standard cineMRI was performed with 10 mm short-axis slices; images were analyzed, epi- and endocardial and PM contours were manually drawn. LV volumes and mass were calculated with and without the inclusion of PMs to the LV mass. Volumes and mass obtained with and without inclusion of PMs were compared using paired samples t-test. Pearson’s correlation coefficient was used to investigate whether PMs mass is related to LV mass.

## Results

Inclusion of PMs to the LV mass, resulted in a significant reduction in LV volumes and an increase in EF (Table 1). PMs-mass for group 1, 2 and 3 was 5.8±1.5 g/m2, 6.8±3.0 g/m2 and 3.9±0.9 g/m2, respectively. The relative contribution of the PMs mass to the absolute LV mass did not differ between groups. However, PMs mass was associated with absolute LV mass in group 1 and 2, r=0.639 (p<.001) and r=0.800 (p<.001), respectively, but not in group 3.

**Table 1:** Impact of in- or exclusion of the papillary muscles to LV mass on LV volumes, mass and EF.

Group 1 (Phenotype+ Genotype+)(n =27)			
	Exclusion	Inclusion	P=

LV EDV (ml/m2)	96.9±15.5	91.4±14.8	<.001
LV ESV(ml/m2)	41.5±13.3	36.9±12.8	<.001
LV EF (%)	57.9±7.2	60.3±7.6	<.001
LV Mass (g/m2)	71.9±23.1	77.8±24.1	<.001

Group 2 (Phenotype+ Genotype-)(n = 36)			

	Exclusion	Inclusion	P=

LV EDV (ml/m2)	90.9±15.5	84.4±13.9	<.001
LV ESV(ml/m2)	37.6±9.9	32.6±8.6	<.001
LV EF (%)	58.8±7.2	61.5±7.5	<.001
LV Mass (g/m2)	78.3±32.0	85.2±34.5	<.001

Group 3(Phenotype- Genotype+)(n = 10)			

	Exclusion	Inclusion	P=

LV EDV (ml/m2)	91.5±9.5	87.8±9.3	<.001
LV ESV(ml/m2)	37.1±7.9	33.7±7.9	<.001
LV EF (%)	59.8±6.0	62.0±6.5	<.001
LV Mass (g/m2)	43.2±10.7	47.0±11.3	<.001

## Conclusions

Inclusion of the papillary muscles to the LV mass significantly changes LV volumes and ejection fraction, in both patients with HCM and preclinical individuals without hypertrophy. Given the prognostic impact of both ejection fraction and LV mass, our data underscore the importance of standardized assessment either with or without inclusion of the papillary muscles to the LV mass.

Given the association between LV mass and papillary muscle mass in patients with HCM, the clinical consequences of assessment with uniform in- or exclusion remains to be determined.

## Funding

This study was funded by the Foundation for Cardiovascular Research of both participating institutes.

